# Impact of Mulberry Consumption on Glycemic Indices and Lipid Profiles: An Umbrella Review

**DOI:** 10.1002/fsn3.72557

**Published:** 2026-11-13

**Authors:** Woorawee Inthachat, Chaowanee Chupeerach, Teerapon Dhippayom, Piya Temviriyanukul

**Affiliations:** ^1^ Food and Nutrition Academic and Research Cluster, Institute of Nutrition Mahidol University Salaya Nakhon Pathom Thailand; ^2^ The Research Unit of Evidence Synthesis, Faculty of Pharmaceutical Sciences Naresuan University Phitsanulok Thailand; ^3^ Advancing Collaboration, Translation, and Innovation for Value in Evidence Synthesis (ACTIVES), Faculty of Pharmaceutical Sciences Naresuan University Phitsanulok Thailand; ^4^ Department of Pharmacotherapy, College of Pharmacy University of Utah Salt Lake City Utah USA

**Keywords:** functional food, human health, meta‐analysis, metabolic syndrome, Morus, umbrella review

## Abstract

The growing worldwide prevalence of type 2 Diabetes Mellitus (T2D) and dyslipidemia requires the identification of effective plant‐based management approaches, including the use of mulberry. However, the clinical evidence about the effects of mulberry on glucose and lipid profiles is inconsistent. This umbrella review studied evidence from five meta‐analyses (MAs) to comprehensively evaluate the impact of mulberry on glycemic and lipid profiles. Systematic searches were performed across major databases with methodological quality and certainty of evidence examined using AMSTAR‐2 and GRADE criteria, respectively. Low‐certainty evidence suggested that mulberry twig alkaloids reduced glycated hemoglobin (HbA1c) in adults with T2D compared with placebo (MD: −0.51%, 95% CI: −0.92 to −0.09). Moderate‐certainty evidence demonstrated that mulberry consumption significantly reduced fasting blood glucose (MD: −34.23 mg/dL, 95% CI: −55.13 to −13.33) and triglycerides (MD: −45.17 mg/dL, 95% CI: −70.86 to −19.49) in adults with T2D. In adults with medical conditions, mulberry also reduced total cholesterol (MD: −13.13 mg/dL; 95% CI: −19.06 to −7.20) and LDL‐C (MD: −8.84 mg/dL; 95% CI: −13.26 to −4.42), with low‐certainty evidence. By contrast, mulberry intake was not associated with changes in fasting plasma insulin and HDL‐C. All five MAs were rated as having critically low methodological quality according to AMSTAR‐2. Therefore, while mulberry may offer potential as a supportive supplement for cardiometabolic management, these findings should be interpreted with caution, are considered preliminary given the critically low methodological quality and the low‐to‐very‐low certainty of evidence for several statistically significant findings, and high‐quality clinical research is needed to strengthen the evidence base.

## Graphical abstract

This umbrella review analyzed five meta‐analyses of randomized controlled trials to evaluate the effect of mulberry (*Morus* spp.) on glycemic control and lipid profiles in adults. Mulberry reduced HbA1c, fasting blood glucose, triglycerides, total cholesterol, and LDL‐C, without significant effects on fasting plasma insulin or HDL‐C. Methodological quality and evidence certainty were assessed with AMSTAR‐2 and GRADE, respectively.

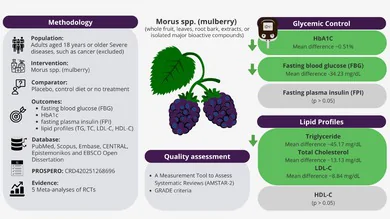

## 1 Introduction

The world is currently dealing with a dual epidemic of Type 2 Diabetes Mellitus (T2D) and dyslipidemia as the leading factors behind heart disease and death (Ballena‐Caicedo et al. 2025; Huang et al. 2025). The International Diabetes Federation (IDF) estimates that by 2045, 783 million people will suffer from diabetes, nearly 12.2% or about 1 in 8 of the global adult population (Sun et al. 2022). T2D is characterized by consistently high blood sugar levels, resulting from a complicated interplay of developing β‐cell failure and different degrees of insulin resistance (Bacha et al. 2024). Such metabolic derangements are often accompanied by changes in lipid profiles, marked by elevated triglycerides (TG), total cholesterol (TC), and low‐density lipoprotein cholesterol (LDL‐C) and low levels of high‐density lipoprotein cholesterol (HDL‐C) (Hirano 2018). In the long term, this metabolic process is associated with extreme oxidative stress and systemic inflammation, leading to serious microvascular and macrovascular complications, including retinopathy, nephropathy, and ischemic heart disease (Caturano et al. 2025; Chawla et al. 2016; Rao et al. 2021). Metformin and synthetic α‐glucosidase inhibitors like acarbose are effective, but they often cause gastrointestinal side effects (Nabrdalik et al. 2022) and may lead to long‐term implications. As a result, interest in using plant‐based therapies and functional foods to help manage these metabolic conditions has recently increased (Srinivasa et al. 2026).

Mulberry ( *Morus alba* L. or *Morus nigra* L.) has become a promising plant‐based alternative option because of its long history in traditional medicine and its unique chemical properties in alleviating several health complications, such as T2D and neurological disorders (Morales Ramos et al. 2021; Suttisansanee et al. 2020; Temviriyanukul et al. 2020). Mulberry contains a wide range of bioactive components such as alkaloids (1‐deoxynojirimycin (DNJ)), flavonoids, polyphenols, and polysaccharides, which interact with and impact a variety of physiological functions involved in glucose and lipid metabolism. These compounds instigate mechanisms that operate by inhibiting α‐glucosidase, a carbohydrate digestive enzyme, enhancing insulin sensitivity, and modulating lipid metabolism pathways (Morales Ramos et al. 2021). However, the effects observed in the glucose and lipid profiles of mulberry are inconsistent, with some reports demonstrating significant improvements while others report negligible effects. For example, Phimarn et al. (2017) showed that mulberry did not affect lipid profiles, while Chen et al. (2022) found a reduction in TC, LDL‐C and TG in the treatment group compared with the control. Such variability highlights the need for a thorough synthesis of the evidence to elucidate the actual therapeutic potential of mulberry.

This umbrella review was conducted to assess and synthesize the evidence from various systematic reviews (SRs) and meta‐analyses (MAs) to determine the evidence regarding the cardiometabolic impacts of mulberry. This methodology facilitated the identification of consistent trends, an assessment of the strength and quality of the evidence, and the clarification of potential research gaps, thereby guiding future clinical and scientific studies (Choi and Kang 2023). Thus, this study evaluated and synthesized the findings from existing SRs and MAs to comprehensively assess the effects of mulberry consumption on glucose and lipid profiles and provide a definitive, evidence‐based framework to guide the standardization of mulberry‐derived functional foods or decision‐making in the clinical management of T2D and dyslipidemia.

## 2 Methods

This review was registered with PROSPERO (CRD420251268696), performed according to the Cochrane Handbook for Systematic Reviews of Interventions version 6.5 (Higgins et al. 2024), and reported in accordance with the PRIOR (Preferred Reporting Items for Overviews of Reviews) statement (Gates et al. 2022). Consistent with the overview design, the objective of the present review was to identify, critically appraise, and synthesize the findings of existing meta‐analyses. Accordingly, all data were extracted and analyzed at the meta‐analysis level, and no re‐analysis of individual randomized controlled trials (RCTs) was performed.

### 2.1 Search Strategy

The CENTRAL, Embase, Epistemonikos, PubMed, Scopus and EBSCO Open Dissertations databases were searched from inception to August 27, 2025. Additionally, backward and forward citation searching was performed using Scopus to identify potentially relevant studies. No language or publication date restrictions were applied during the search process, with the full search strategy for each database shown in Tables S1–S6.

### 2.2 Study Selection

The eligibility criteria were defined according to the Population, Intervention, Comparator, Outcomes, and Study design (PICOS) framework. The population (*P*) comprised adults aged 18 years or older. Studies involving participants with severe diseases, such as cancer, were excluded. Eligible interventions (*I*) included the consumption of any form or part of *Morus* spp. (mulberry), including whole fruit, leaves, root bark, extracts, or isolated major bioactive compounds administered as a single preparation. Studies evaluating multi‐ingredient formulations were also eligible provided that the amount or concentration of mulberry, its extract, or its principal bioactive compounds was clearly quantified. The comparator (*C*) could be any control condition, including placebo, control diet, no treatment, or active comparators such as hypoglycemic drug therapy. Outcomes (*O*) were fasting blood glucose (FBG), HbA1c, fasting plasma insulin (FPI), and lipid profiles (TG, TC, LDL‐C, HDL‐C). Only systematic reviews and meta‐analyses of RCTs were eligible for inclusion.

Studies with (1) meta‐analyses that provided insufficient or inadequate data for the selection process and/or quantitative synthesis, and (2) those that involved subjects with severe diseases (e.g., cancer) were excluded. Lists of included and excluded MAs are provided as Table S7. When two or more MAs addressed the same review question (i.e., the same population, intervention scope, comparator, and outcome set) and drew on the same primary RCTs, only the most recent or most comprehensive MA was included. All other MAs were retained, including those whose review questions overlapped, since excluding an MA that drew on a different set of primary RCTs would have discarded primary evidence not captured by the others.

Because this approach allowed individual RCTs to contribute to more than one included MA, we assessed overlap by cross‐tabulating the primary RCTs against the MAs at the review level, and by recording which outcome‐specific pooled estimate each RCT contributed to. In addition, this umbrella review used the pooled estimates as reported in the included MAs and did not re‐pool primary‐study data across MAs, no RCTs were counted more than once within any single pooled estimate presented in this review. However, pooled estimates from different MAs are not statistically independent when they include the same primary RCTs. Two authors (W.I. and C.C.) independently reviewed titles and abstracts and assessed the full texts of potentially eligible articles. Discrepancies were addressed and resolved by a third reviewer (P.T.).

### 2.3 Data Extraction and Quality Assessment

The data from MAs were independently extracted by W.I. and P.T., including (1) general information (authors, year, location), (2) participants, (3) intervention and comparators, (4) outcomes of measures, and (5) funding (if available).

The MAs were evaluated for their methodological quality or risk of bias using A Measurement Tool to Assess Systematic Reviews (AMSTAR‐2) (Shea et al. 2017), which was performed independently by W.I. and P.T. Any discrepancies during data extraction and methodological quality assessment were resolved by the third author (C.C.). The results of AMSTAR‐2 were used to guide the evaluation of evidence quality and the reporting of this review.

### 2.4 Statistical Analysis

The pooled effect estimates (e.g., mean difference (MD)), 95% confidence intervals (CIs), and measures of heterogeneity (*I* ^2^) were extracted for each association, as reported in each included MA. One meta‐analysis (Chen et al. 2025) reported estimates as SMD. Because SMD are less clinically interpretable (Higgins et al. 2024), we re‐expressed its results as MD on the original (mg/dL) scale, using the group sample sizes, means, and standard deviations (SD) reported within that meta‐analysis together with a random‐effects model. The *p*‐value of < 0.05 considered statistically significant.

### 2.5 Evidence Grading

The certainty of evidence for each outcome was evaluated using the GRADE criteria (Grading of Recommendations, Assessment, Development, and Evaluations) (Guyatt et al. 2008) through GRADEpro GDT (McMaster University, Canada). This encompassed five domains as risk of bias, inconsistency, indirectness, imprecision, and publication bias. The strength of evidence supporting each association, according to GRADE criteria, was categorized as high, moderate, low, or very low. Two authors (W.I. and P.T.) independently evaluated the GRADE criteria, with C.C. serving as the third author to resolve any discrepancies through group discussion.

## 3 Results

### 3.1 Study Selection

The PRISMA flow diagram illustrating the database search process is presented in Figure 1. The comprehensive database search strategy identified 26 eligible studies, while 416 eligible studies were obtained via other search methods, including reference searching and citation searching. After screening the titles and abstracts, eight studies were considered suitable for full‐text review. Five MAs (Chen et al. 2025, 2022; Cui et al. 2023; Jeong et al. 2022; Phimarn et al. 2017) were included in this umbrella review, while three were excluded due to the absence of relevant interventions or outcomes of interest.

**FIGURE 1:**
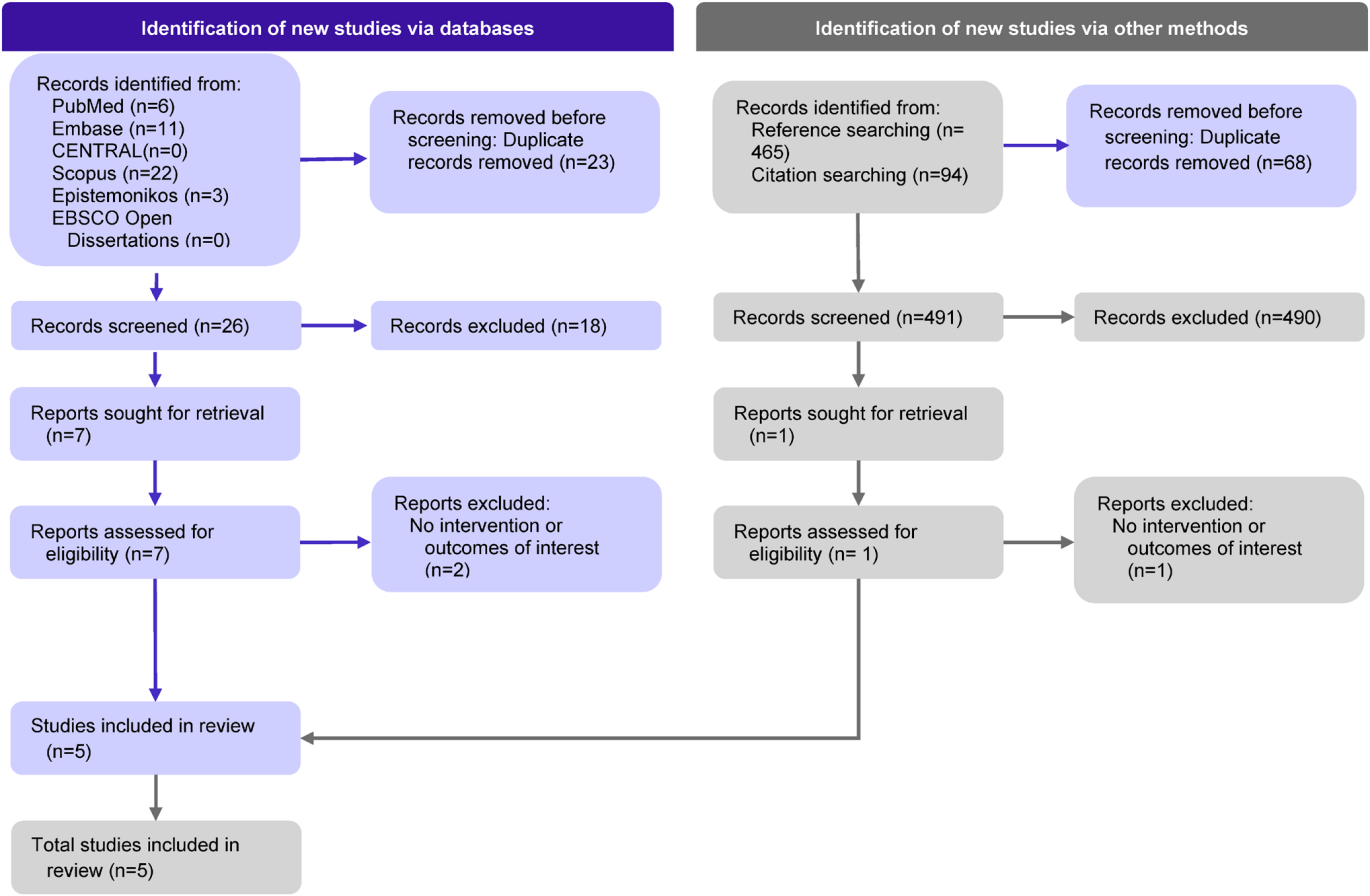
The PRISMA flow diagram of article selection process.

### 3.2 Study Characteristics and Quality Assessment

Table 1 presents the characteristics of the five MAs utilized in this umbrella review, published between 2017 and 2025, consisting of 48 RCT inclusions involving 2903 subjects when summed across MAs (Chen et al. 2025, 2022; Cui et al. 2023; Jeong et al. 2022; Phimarn et al. 2017). A total of 28 unique comparisons were identified as shown in Table 2 (6 comparisons for HbA1c and FBG, 2 comparisons for FPI, 4 comparisons for TC and TG, and 3 comparisons for LDL‐C and HDL‐C). Out of these 28 comparisons, 9 were statistically significant, including comparisons in HbA1c, FBG, TG, TC, and LDL‐C.

**TABLE 1:** Summary characteristics of the included meta‐analyses (MAs).

Author and year	Location	Population	Intervention	Comparator	Outcomes	Duration	No. of studies (no. of subjects)	AMSTAR‐2	Funding
Chen et al. (2025)	China	T2D	Mulberry twig alkaloids 50–100 mg, TID (3)^#^ Mulberry twig alkaloid 50–100 mg, TID + acarbose placebo (2)	Placebo (2)^#^ Acarbose 50–100 mg, TID (2) Metformin 0.5–1 g, BID (1)	HbA1c, FBG, 2hPG, TG, TC, LDL‐C, HDL‐C	12 weeks to 24 weeks	5 (821)^$^	Critically low	University of Hong Kong
Cui et al. (2023)	China	1. Healthy adults 2. T2D and pre‐T2D 3. Hyperlipidemia 4. Obesity	Mulberry leaf 3 g/day (2) Mulberry leaf tea 5 g (1) Mulberry leaf extract 200–2500 mg (amount of DNJ 12–36 mg) (8) DNJ‐rich powder 1.2 g (1)	NR (12)	HbA1c, FBG and FPI	4 weeks to 24 weeks	12 (651)	Critically low	China Government
Chen et al. (2022)	Iran	T2D Dyslipidemia Impaired glucose metabolism T2D nephropathy Osteoarthritis CVD	Mulberry leaf powder 280 mg, TID or 3000 mg, TID (3) Mulberry leaf extract or 300–1000 mg/day (3) Freeze‐dried mulberry 45 g (1) Mulberry twig alkaloid (SZ‐A) 100 mg (1) UP1306 100 mg (1) DNJ 10–150 mg/day (3)	1. Placebo (7) 2. Glibenclamide 5 g (1) 3. Usual diet (3) 4. Acarbose 100 mg (1)	HbA1c, FBG, FPI, TC, TG, LDL‐C and HDL‐C	4 weeks to 48 weeks	12 (786)	Critically low	NR
Jeong et al. (2022)	Republic of Korea	FBS (100–140 mg/dL) or PPG 120 min between 140 and 199 mg/dL Healthy adults T2D	Diet education + mulberry powder 4.6 g, TID (DNJ 12 mg per dose) (1) Mulberry powder 1.2 g, TID (amount of DNJ 18 mg) (1) Mulberry tablet 3.6 mg, TID (1) Mulberry capsule 0.5 g, BID (1) Mulberry capsule 6 mg‐1 g, TID (2)	Diet education + no mulberry (1) No mulberry (1) Placebo (3) Cellulose (1)	HbA1c, FBG, FPI, TC, TG, LDL‐C and HDL‐C	4 weeks to 12 weeks	6 (209)^†^	Critically low	Korea Government
Phimarn et al. (2017)	Thailand	Healthy adults T2D Impaired glucose tolerance Hypercholesterolemia statin not required or statin intolerant subjects	DNJ 0.254–18 mg (7) Mulberry + banana + Korean red ginseng (1) Mulberry tea 70 mL or 2 g, TID (2) Mulberry + other herbal (1) Mulberry extract 350 mg, 2 capsules, TID (1) Combination B* (1)	1. Placebo (12) 2. Combination A* (1)	PPG 30, 60, 90, 120 min, HOMA‐IR, FBS, HbA1c, TC, TG, LDL‐C, and HDL‐C	1 day to 180 days	13 (436)	Critically low	NR

*Note:* ^#^Values in parentheses indicate the number of RCTs included for each intervention and comparator. ^$^This MA included nine RCTs; however, only five were included because the remaining four evaluated mulberry as an adjunct to antidiabetic drugs. ^†^This MA included 13 RCTs; however, only six were included because the remaining seven evaluated outcomes that were not relevant to the inclusion criteria and (*) Combination A: Policosanol 10 mg, red yeast rice 200 mg, berberine 500 mg, astaxanthin 0.5 mg, folic acid 200 μg, coenzyme Q10 2 mg; Combination B: Berberine 531.25 mg, red yeast rice 220 mg, leaf extract of *M. alba* 200 mg.

Abbreviations: 2hPG, 2‐h Plasma Glucose; BID, two times a day; CVD, coronary heart disease; DNJ, 1‐deoxynojirimycin; HOMA‐IR, Homeostasis model assessment of insulin resistance; NR, not report; PPG 120 min, postprandial plasma glucose at 120 min; PPG 30 min, postprandial plasma glucose at 30 min; PPG 60 min, postprandial plasma glucose at 60 min; PPG 90 min, postprandial plasma glucose at 90 min; TID, three times a day.

**TABLE 2:** Estimates of glycemic indices and lipid profiles following consumption of various mulberry‐based preparations.

Author	Heath status	Intervention	Comparator	No. of studies (no. of subjects)	ES (95% CI)	*I* ^2^ (*p*)	ROB tool	Average risk of bias of included studies	GRADE assessment
HbA1c (%)									
Chen et al. (2025) (study A)	T2D	Mulberry twig alkaloids	Placebo	2 (181)	−0.51 (−0.92 to −0.09)^#^	78.05% (0.03)	ROB‐2	S	Low
Chen et al. (2025) (study B)	T2D	Mulberry twig alkaloids + acarbose placebo	Acarbose^†^	2 (579)	−0.06 (−0.21 to 0.10)^#^	0% (0.77)	ROB‐2	L	Low
Cui et al. (2023) (study B)	Healthy adults Various medical conditions	Various mulberry‐based preparations	NR	11 (590)	−0.27 (−0.52 to −0.02)^$^	92% (0.00)	ROB‐1	S	Very low
Chen et al. (2022)	Various medical conditions	Various mulberry‐based preparations	Mixed comparators^†^	5 (196)	−0.55 (−1.08 to −0.02)	91.3% (0.00)	ROB‐1	S	Very low
Jeong et al. (2022)	Healthy adults Various medical conditions	Diet education + mulberry Various mulberry‐based preparations	Mixed comparators^†^	4 (159)	−0.05 (−0.18 to 0.08)	0% (0.75)	ROB‐1	S	Moderate
Phimarn et al. (2017)	Healthy Various medical conditions	Various mulberry‐based preparations	Placebo + combination A*^,†^	5 (274)	−0.05 (−0.19 to 0.09)	0% (0.95)	Jadad scale and ACROBAT	L	Low
FBG (mg/dL)									
Chen et al. (2025) (study A)	T2D	Mulberry twig alkaloids	Placebo	1 (30)	−34.23 (−55.13 to −13.33)^#^	NA	ROB‐2	S	Moderate
Chen et al. (2025) (study B)	T2D	Mulberry twig alkaloids + acarbose placebo	Acarbose or metformin^†^	3 (599)	−1.26 (−7.21 to 4.68)^#^	0% (0.42)	ROB‐2	S	Very low
Cui et al. (2023)	Healthy adults Various medical conditions	Various mulberry‐based preparations	NR	12 (615)	−8.47 (−15.13 to −1.80)	96% (0.00)	ROB‐1	S	Very low
Chen et al. (2022)	Various medical conditions	Various mulberry‐based preparations	Mixed comparators^†^	5 (239)	−7.50 (−19.58 to 4.59)	86% (0.00)	ROB‐1	S	Very low
Jeong et al. (2022)	Healthy adults Various medical conditions	Diet education + mulberry Various mulberry‐based preparations	Mixed comparators^†^	3 (142)	−0.79 (−4.56 to 2.98)	0% (0.37)	ROB‐1	S	Very low
Phimarn et al. (2017)	Healthy Various medical conditions	Various mulberry‐based preparations	Placebo + combination A^†^	7 (346)	−4.50 (−9.91 to 0.90)^$^	69% (0.003)	Jadad scale and ACROBAT	S	Very low
FPI (μIU/mL)									
Cui et al. (2023)	Healthy adults Various medical conditions	Various mulberry‐based preparations	NR	5 (214)	−0.58 (−2.78 to 1.62)	55% (0.06)	ROB‐1	S	Very low
Chen et al. (2022)	Various medical conditions	Various mulberry‐based preparations	Placebo	3 (179)	0.13 (−1.29 to 1.55)	0% (0.859)	ROB‐1	S	Very low
Triglycerides (mg/dL)									
Chen et al. (2025) (study A)	T2D	Mulberry twig alkaloids	Placebo	1 (30)	−45.17 (−70.86 to −19.49)^#^	NA	ROB‐2	S	Moderate
Chen et al. (2025) (study B)	T2D	Mulberry twig alkaloids + acarbose placebo	Acarbose^†^	1 (36)	−18.60 (−81.49 to 44.29)^#^	NA	ROB‐2	L	Low
Chen et al. (2022)	Various medical conditions	Various mulberry‐based preparations	Mixed comparators^†^	7 (416)	−19.67 (−30.13, −9.22)	58.9% (0.023)	ROB‐1	S	Very low
Phimarn et al. (2017)	Various medical conditions	Various mulberry‐based preparations	Placebo + combination A^†^	4 (158)	−17.71 (−37.20 to 1.77)^$^	0% (0.41)	Jadad scale and ACROBAT	L	High
Cholesterol (mg/dL)									
Chen et al. (2025) (study A)	T2D	Mulberry twig alkaloids	Placebo	1 (30)	−10.83 (−30.16 to 8.51)^#^	NA	ROB‐2	S	Low
Chen et al. (2025) (study B)	T2D	Mulberry twig alkaloids + acarbose placebo	Acarbose^†^	1 (36)	2.71 (−23.20 to 28.62)^#^	NA	ROB‐2	L	Low
Chen et al. (2022)	Various medical conditions	Various mulberry‐based preparations	Mixed comparators^†^	7 (416)	−13.13 (−19.06, −7.20)	46.2% (0.083)	ROB‐1	S	Low
Phimarn et al. (2017)	Various medical conditions	Various mulberry‐based preparations	Placebo + combination A^†^	4 (158)	−6.57 (−23.98 to 10.44)^$^	65% (0.03)	Jadad scale and ACROBAT	L	Moderate
LDL‐C (mg/dL)									
Chen et al. (2025)	T2D	Mulberry twig alkaloids + acarbose placebo	Acarbose^†^	1 (36)	−5.80 (−22.04 to 10.44)^#^	NA	ROB‐2	L	Moderate
Chen et al. (2022)	Various medical conditions	Various mulberry‐based preparations	Mixed comparators^†^	7 (416)	−8.84 (−13.26, −4.42)	27.7% (0.217)	ROB‐1	S	Low
Phimarn et al. (2017)	Various medical conditions	Various mulberry‐based preparations	Placebo + combination A^†^	4 (158)	−4.25 (−20.11 to 11.60)^$^	69% (0.02)	Jadad scale and ACROBAT	L	Very low
HDL‐C (mg/dL)									
Chen et al. (2025)	T2D	Mulberry twig alkaloids + acarbose placebo	Acarbose^†^	1 (36)	−2.32 (−6.96 to 2.32)^#^	NA	ROB‐2	L	High
Chen et al. (2022)	Various medical conditions	Various mulberry‐based preparations	Mixed comparators^†^	7 (416)	1.51 (−0.40, 3.42)	21.4% (0.266)	ROB‐1	S	Low
Phimarn et al. (2017)	Various medical conditions	Various mulberry‐based preparations	Placebo + combination A^†^	4 (158)	0.00 (−3.87 to 4.25)	65% (0.03)	Jadad scale and ACROBAT	L	Very low

*Note:* The pooled effect estimates are mean difference (MD). ^*^Combination A: Policosanol 10 mg, red yeast rice 200 mg, berberine 500 mg, astaxanthin 0.5 mg, folic acid 200 μg, coenzyme Q10 2 mg; ^#^For this MA, which reported SMD, we re‐extracted sample sizes, means, and standard deviations (SD) reported within the meta‐analysis (Chen et al. 2025), converted the outcome values to mg/dL, and re‐pooled the data using a random‐effects model to obtain MD, 95% CI and *I* ^2^. ^$^Cui et al. (2023) and Phimarn et al. (2017) reported glucose and lipids in mmol/L; we converted them to mg/dL using the same factors applied to Chen et al. (2025) (glucose × 18.016; TC, LDL‐C and HDL‐C × 38.67; TG × 88.57). ^†^Pooled estimate includes one or more trials using an active comparator (acarbose, metformin, glibenclamide, or a berberine‐containing combination product). These estimates should be interpreted relative to the comparator(s) included, rather than as effects versus placebo. Active‐comparator use does not itself imply underestimation or overestimation. Comparisons between multi‐ingredient formulations that also differ in other components or doses do not isolate the effect of mulberry.

Abbreviations: NA, not available; NR, no report.

Because individual RCTs could be included in more than one MA, we assessed overlap by cross‐tabulating the primary RCTs across the five included MAs and recording the outcome‐specific pooled estimates to which each RCT contributed (Table S8). The 48 report‐level inclusions corresponded to 34 unique RCTs; 9 RCTs appeared in more than one MA, of which 6 contributed to pooled estimates of the same outcome in two or more MAs. Except for the MA by Chen et al. (2025), for which we re‐pooled the reported SMD estimates as MDs within that MA (Chen et al. 2025), this umbrella review used the pooled estimates as reported in the included MAs and did not combine primary‐study data across MAs. Thus, our analysis did not introduce duplicate inclusion of any RCT within a pooled estimate. However, in the original MA by Phimarn et al. (2017), the 12‐ and 24‐week results from one trial by Kim et al. (2012) were entered as separate comparisons in the FBG and HbA1c analyses, resulting in the same participants contributing twice to those pooled estimates. Moreover, pooled estimates from different MAs are not independent when they include the same primary RCTs.

The participants were healthy subjects and individuals with various health conditions, primarily T2D, dyslipidemia, and obesity. A smaller proportion were obese with borderline T2D and osteoarthritis, as well as patients with T2D and nephropathy. For clarity of interpretation, these populations were collectively categorized as individuals with medical conditions. The reviewed studies focused on mulberry‐derived products (e.g., mulberry leaf or twig extracts, powders, teas, capsules, DNJ‐containing products, and combination products) compared with a placebo, standard diet, or T2D medicine such as acarbose, metformin, glibenclamide, or other combination regimens.

The duration of interventions varied from 1 day to 48 weeks, with most of the trials implementing a treatment period between 4 and 24 weeks. Almost all the studies analyzed FBG, HbA1c, and lipid profiles, while only two examined FPI.

All the included MAs were rated as having critically low methodological quality according to AMSTAR‐2 (Table 1 and Table S9), mainly due to the presence of multiple critical domain flaws, including no justification of the excluded studies and failure to incorporate the risk of primary study bias into the interpretation of the results. Conversely, all the studies demonstrated rigor in defining the research questions, conducting study selection and data extraction in duplicate, utilizing the appropriate statistical pooling techniques, and investigating publication bias, indicating acceptable practices in these aspects.

### 3.3 Effects on Hemoglobin A1c (HbA1c)

Five MAs reported the effects on HbA1c following the consumption of various mulberry‐based preparations among individuals with (i) T2D (Chen et al. 2025), (ii) healthy individuals with medical conditions (Cui et al. 2023; Jeong et al. 2022; Phimarn et al. 2017), and (iii) those solely with medical conditions (Chen et al. 2022) (Table 2). In adults with T2D, mulberry twig alkaloids significantly reduced HbA1c compared to the placebo across two RCTs (*n* = 181) (MD −0.51%, 95% CI −0.92 to −0.09; *I* ^2^ = 78.05%), but showed no statistically significant difference compared with acarbose (Chen et al. 2025). Both analyses were classified as low certainty of evidence (Table 2). In healthy individuals and those with medical conditions, Cui et al. (2023) reported a statistically significant reduction in HbA1c (MD −0.27%, 95% CI −0.52 to −0.02; *I* ^2^ = 92%), whereas Jeong et al. (2022) found no significant effect (MD −0.05%, 95% CI −0.18 to 0.08; *I* ^2^ = 0%) (Cui et al. 2023; Jeong et al. 2022). The certainty of evidence was very low for Cui et al. (2023) and moderate for Jeong et al. (2022). Likewise, in healthy individuals and those with medical conditions, Phimarn et al. (2017) reported no significant effect on HbA1c, with low certainty. Among subjects with medical conditions, Chen et al. (2022) found a modest but statistically significant reduction in HbA1c from 5 RCTs (*n* = 196) (MD −0.55%, 95% CI −1.08 to −0.02; *I* ^2^ = 91.3%), with very low certainty of evidence (Table 2).

### 3.4 Effects on Fasting Blood Glucose (FBG)

In T2D populations, comparisons of mulberry twig alkaloids versus a placebo based on an RCT (*n* = 30) revealed a significant reduction in FBG (MD: −34.23 mg/dL, 95% CI −55.13 to −13.33), with moderate certainty of evidence (Chen et al. 2025) (Table 2 and Table S10). By contrast, no statistically significant difference in FBG was observed between mulberry twig alkaloids and active comparators (acarbose or metformin) (Chen et al. 2025). For healthy individuals and those with medical conditions, the evidence was mixed. One pooled analysis identified a significant reduction in FBG (MD: −8.47 mg/dL, 95% CI −15.13 to −1.80, with very low certainty of evidence) (Cui et al. 2023), whereas other comparisons in similar populations found no significant effects (very low certainty of evidence) (Jeong et al. 2022). Similarly, in analyses restricted to subjects with medical conditions (Chen et al. 2022), no significant changes in FBG were observed.

### 3.5 Effects on Fasting Plasma Insulin (FPI)

Two MAs with 8 RCTs (*n* = 393) (Chen et al. 2022; Cui et al. 2023) reported FPI following consumption of various mulberry‐based preparations among healthy adults and individuals with medical conditions, with no statistically significant effects observed in either population (Table 2).

### 3.6 Effects on Lipid Profiles (TC, TG, LDL‐C and HDL‐C)

Specific lipid profile comparisons showed significant changes, especially in TG, TC and LDL‐C. For T2D patients, mulberry twig alkaloids reduced TG by a significant extent relative to a placebo, with moderate certainty of evidence (MD: −45.17 mg/dL, 95% CI −70.86 to −19.49) (Chen et al. 2025). In subjects with medical conditions, results were divergent. One study by Chen et al. (2022) identified statistically significant lower TG (MD: −19.67 mg/dL, 95% CI: −30.13, −9.22, very low certainty of evidence) and LDL‐C (MD: −8.84 mg/dL, 95% CI: −13.26, −4.42, low certainty of evidence) (Chen et al. 2022). In contrast, Phimarn et al. (2017) found no statistically significant differences in TG (MD: −17.71 mg/dL, 95% CI: −37.20 to 1.77; high certainty of evidence) or LDL‐C (MD: −4.25 mg/dL, 95% CI: −20.11 to 11.60; very low certainty of evidence) in populations with medical conditions. Chen et al. (2022) also reported a statistically significant reduction in TC (MD: −13.13 mg/dL, 95% CI: −19.06 to −7.20), with low certainty of evidence. The other TC comparisons and all HDL‐C comparisons showed no statistically significant differences from their respective controls (Table 2).

## 4 Discussion

This umbrella review summarized previous systematic reviews and meta‐analyses to elucidate the impact of mulberry (*Morus* spp.) on healthy participants or individuals with medical conditions, strengthened by a rigorous assessment of the certainty of evidence using GRADE criteria. Data were collected from five meta‐analyses (Chen et al. 2025, 2022; Cui et al. 2023; Jeong et al. 2022; Phimarn et al. 2017). Moderate‐certainty evidence supported reductions in FBG and TG in patients with T2D, whereas low‐certainty evidence suggested a reduction in HbA1c with mulberry twig alkaloids compared with placebo in T2D subjects. In broader populations, including healthy adults and individuals with other medical conditions, findings for HbA1c were inconsistent, and the statistically significant reductions were supported by very‐low‐certainty evidence.

The findings on glycemic control indicated that the effects of mulberry consumption varied according to population characteristics and clinical status. For HbA1c, the evidence suggested that the effect of mulberry intake depended on the clinical features of the study population. The HbA1c reduction reported for mulberry twig alkaloids versus placebo was based on two RCTs involving 181 adults with T2D and was supported by low‐certainty evidence (Chen et al. 2025). By contrast, meta‐analyses by Cui et al. (2023) and Jeong et al. (2022), which included both healthy subjects and individuals with medical conditions, showed inconsistent findings, ranging from significant to insignificant effects (Cui et al. 2023; Jeong et al. 2022). These variabilities were due to the high heterogeneity shown in mixed‐population analyses, especially in Cui et al. (2023) (Cui et al. 2023), raising concerns regarding the robustness and generalizability of HbA1c reductions in the non‐diabetic group.

A similar pattern of population‐specific efficacy was observed in FBG. The most compelling evidence related to subjects with T2D. Chen et al. (2025) demonstrated a significant reduction of FBG compared to the placebo, indicating a role for mulberry‐derived alkaloids as an adjuvant therapy for T2D patients. By contrast, wide variation was apparent among heterogeneous populations of healthy adults and mild medical patients. Cui et al. (2023) demonstrated a statistically significant reduction in FBG, while Phimarn et al. (2017) observed no significant difference within a comparable mixed population. Differences in comparator treatments may explain the variation across meta‐analyses. However, this review could not determine whether comparator choice, population characteristics, or other study‐level differences primarily explained the different findings. The meta‐analysis by Phimarn et al. (2017) included one trial comparing a combination product containing mulberry with an active formulation with berberine and red yeast rice. Since both study arms contained several bioactives, the difference observed is reflective of the relative effect of the whole formulations and does not clarify the contribution of mulberry (Phimarn et al. 2017). In general, trials with active comparators estimate the effect of mulberry versus the treatment used as the comparator, instead of its effect versus placebo. An active comparator is not a source of inherent bias for this relative treatment estimate, but the results should be interpreted considering the interventions that were compared. Sensitivity analyzes excluding active‐comparator trials were not performed in this umbrella review, and thus their impact on the pooled estimates could not be quantified. Future meta‐analyses should consider placebo‐controlled and active‐controlled trials separately and separate single‐ingredient mulberry intervention from multi‐ingredient formulations. A broader discrepancy between the robust results seen in T2D patients versus the variable results in mixed populations also highlighted a fundamental baseline effect in blood biochemistry. Individuals with pronounced baseline hyperglycemia, such as those evaluated by Chen et al. (2025), possessed a greater physiological capacity to respond to the α‐glucosidase inhibitory properties of mulberry than normoglycemic or mildly impaired subjects, where physiological homeostasis limited the potential for further glucose reduction.

None of the included MAs, reporting on FPI, found a statistically significant effect (Chen et al. 2022; Cui et al. 2023), suggesting that the primary mechanism of action of mulberry may not rely on the stimulation of insulin secretion, but may be focused on pre‐absorptive carbohydrate modulation. However, this interpretation differed from the evidence obtained from animal models, with administration of DNJ to diabetic mice (20, 40, and 80 mg/kg via intravenous injection for 4 weeks) resulting in dose‐dependent improvements in insulin resistance, enhanced insulin sensitivity, and reduced blood glucose levels (Liu et al. 2015). This result was due to the use of intravenous DNJ at pharmacological doses in mice, which bypassed the intestinal lumen and revealed systemic insulin‐signaling effects not captured to the same extent in oral, food‐based human interventions.

The effects of mulberry on lipid profiles were most inconsistent among the included MAs. For example, Chen et al. (2022) reported a significant reduction of TG in subjects with medical conditions, whereas Phimarn et al. (2017) reported a non‐significant effect. These different findings are consistent with a dose‐ and formulation‐dependent threshold effect, whereby mulberry produces significant lipid‐lowering effects only above a certain intake or with particular standardized preparations, and little or no effect at lower doses or with non‐standardized products. Our results also showed a significant reduction in TC and LDL‐C, especially in certain conditions (Chen et al. 2022). For example, a daily intake exceeding 300 mg of mulberry was suggested as necessary to achieve favorable effects on serum lipids (Chen et al. 2022). By contrast, Phimarn et al. (2017) did not observe a significant lipid‐lowering effect, possibly explained by the lower mean dosage and the lack of standardization of the mulberry preparations used in the primary studies.

There is evidence that the variety of mulberry preparation might influence glycemic and lipid outcomes. However, this could not be formally evaluated because the included MAs pooled different types of mulberry preparations rather than performing analyses by formulation. The most consistent improvements in glycemic outcomes were reported for standardized preparations with DNJ, mainly mulberry twig alkaloids. In T2D patients, these preparations reduced FBG and TG with moderate certainty (single RCT) and HbA1c with low certainty (Chen et al. 2025) in line with the hypothesis that DNJ is an α‐glucosidase inhibitor that can decrease carbohydrate absorption. The crude leaf powders, teas, and non‐standardized leaf extracts, which were generally given at lower or variable amounts of this plant, were less frequently effective for glycemic or lipid effects (Cui et al. 2023; Jeong et al. 2022; Phimarn et al. 2017). For lipid outcomes, significant reductions in TG, TC, and LDL‐C were reported primarily with higher dose interventions, with an MA suggesting daily levels greater than 300 mg may be needed and lower dose or non‐standardized preparations having no effect (Chen et al. 2022; Phimarn et al. 2017). In the context of combination products, the interpretation of studies using combination products is more complicated as bioactive ingredients such as berberine and red yeast rice can affect glucose and lipid metabolism independently. So mulberry‐treated studies are not based solely on mulberry alone and can overestimate or underestimate their effect (Phimarn et al. 2017). Taken together, these findings suggest that the efficacy of mulberry depends not only on the plant itself but also on the formulation, degree of standardization, and dose.

Several cellular mechanisms have been proposed, based on preclinical (animal and in vitro) studies rather than the human meta‐analyses included in this review, to explain how mulberry alters lipid profiles including (i) initiation of the adenosine monophosphate‐activated protein kinase (AMPK) pathway, resulting in increased beta‐oxidation of free fatty acids and the suppression of lipid accumulation (Tsuduki et al. 2009), (ii) the suppression of lipogenic enzymes and decreased lipogenesis via sterol regulatory element‐binding proteins 1c (SREBP‐1c) and fatty acid synthase (Ann et al. 2015; Duangjai et al. 2011), and (iii) the activation of peroxisome proliferator‐activated receptor alpha (PPARα) and peroxisome proliferator‐activated receptor gamma (PPARγ) involved in cholesterol metabolism (Kobayashi et al. 2015). However, lipid outcomes were inconsistent across the included MAs. Significant reductions in TC and LDL‐C, both supported by low‐certainty evidence, were reported only by Chen et al. (2022) in individuals with medical conditions (Chen et al. 2022), TG was reduced in Chen et al. (2022) and in the single T2D trial in Chen et al. (2025), and no MA showed a change in HDL‐C. The current evidence therefore cannot determine whether mulberry acts mainly through pathways such as AMPK activation or suppression of lipogenic enzymes, or through broader inhibition of cholesterol biosynthesis, as seen with HMG‐CoA reductase inhibitors (Stancu and Sima 2001). Importantly, these mechanistic pathways (AMPK activation, SREBP‐1c suppression, and PPARα/γ modulation) are derived from preclinical animal and in vitro studies, and none of the included meta‐analyses provided direct mechanistic evidence in humans. They should therefore be regarded as hypothesized biological explanations that require confirmation in human studies, whereas the clinical evidence synthesized in this review is limited to the observed effects on FBG, HbA1c, TG, TC, and LDL‐C.

To explain the results of this umbrella review in a broader context, we also considered recent primary studies on mulberry. Although these studies were not eligible for inclusion because they were individual RCTs, animal experiments, in vitro and in silico studies and not meta‐analyses of RCTs, they provide some context for our analysis. In particular, they address three critical issues in our synthesis: the combined effect of mulberry on glucose and lipid metabolism, the confounding effect of other active ingredients in combination products, and the biological processes behind its cardiometabolic effects. In a multicenter randomized controlled trial with 359 cardiometabolic patients, monacolin K plus *M. alba* combined reduced FBG, HbA1c and the HOMA index significantly more than Monacolin K alone and hence the use of *M. alba* may be beneficial for glucose‐ and lipid‐lowering in combination formulation (Pacella et al. 2025). Similarly, the LopiGLIK study suggested that ingredients such as *M. alba* , rather than monacolin K content alone, may contribute to the lipid‐lowering efficacy of multi‐component nutraceuticals (Izzo et al. 2024). However, these combination formulations also highlight the difficulty of attributing the observed effects specifically to mulberry because of potential confounding by co‐administered bioactive compounds, such as berberine and red yeast rice reported in Phimarn et al. (2017). Mechanistic evidence from preclinical studies further suggests potential biological pathways. A mulberry‐ and *Hippophae*‐based beverage promoted weight loss in rats by modulating white adipose tissue PPARγ and FGFR1 signaling (Zhou et al. 2024). The combination of mulberry leaf and corn silk inhibited α‐amylase and α‐glucosidase activity in vitro and reduced postprandial glucose responses in persons with prediabetes (Sun et al. 2025), findings that are consistent with the proposed α‐glucosidase inhibitory activity discussed in this review. Moreover, phytochemical profiling of *M. alba* bark with *Pinus densiflora* and methyl gallate showed a considerable degree of compositional complexity, highlighting the need for the standardization of mulberry preparations (Abbas et al. 2025); however, this study was on antimicrobial, antioxidant and anti‐inflammatory activities rather than on glycemic or lipid parameters. These studies provide complementary mechanistic and clinical context, but do not change the conclusions drawn from the evidence synthesized in this umbrella review.

### 4.1 Limitations of the Current Evidence

This umbrella review had some important limitations within the existing body of evidence that warrant cautious interpretation of the findings. (i) The included meta‐analyses pooled structurally and pharmacologically diverse mulberry preparations, covering leaf, twig, and fruit extracts, powders, teas, capsules, DNJ‐containing products, and combination formulations, within the same analyses. Because these preparations differ substantially in bioactive composition, dose, and delivery, this clinical heterogeneity is a major source of the observed statistical heterogeneity and limits both the interpretability of the pooled effect sizes and their generalizability to any single mulberry product. Differences in comparator treatments further limit comparability across estimates. Active‐controlled comparisons address relative effects against the specified treatment, while comparisons of formulations that differ in multiple active components cannot isolate mulberry‐specific effects. The summary estimates should therefore be viewed as broad indications of the direction of effect rather than precise, product‐specific treatment effects; this uncertainty was formally captured in the GRADE assessment through downgrading for heterogeneity. (ii) Because risk of bias is one of the domains evaluated in the GRADE framework, differences in the risk‐of‐bias tools used across the included meta‐analyses may have influenced the certainty ratings, and the ratings are therefore not strictly comparable across our effect estimates. More rigorous tools (e.g., RoB 2) tend to identify methodological concerns more often and may therefore increase the likelihood of downgrading the certainty of evidence, whereas less stringent tools (e.g., the Jadad scale) may produce more favorable assessments of the primary studies and consequently be less likely to result in downgrading. Certainty may therefore be over‐rated for estimates derived from meta‐analyses that used less comprehensive tools, relative to those that used RoB 2. In addition, because we assessed the methodological quality of the included meta‐analyses using AMSTAR‐2 rather than re‐evaluating every primary trial with a single standardized risk‐of‐bias instrument, the variability in the risk‐of‐bias tools applied across the included reviews should be considered a limitation of our certainty assessment. (iii) The methodological quality of the included evidence was critically low, with all five included MAs rated as critically low quality based on AMSTAR‐2. These deficiencies significantly reduced confidence in the robustness of the synthesized estimates. (iv) The high heterogeneity suggested that a particular pooled effect size may not indicate wide variations in responses across varied populations, interventions, dosages, and study methodologies. Consequently, the applicability of summary estimates to specific subgroups remained questionable. (v) The short intervention durations reduced the interpretability of the evidence. Most of the included randomized controlled trials were performed for relatively brief durations, usually between 4 and 24 weeks, which were too short to assess long‐term clinical results, including cardiovascular outcomes or diabetes‐associated complications, (vi) although some outcomes showed statistically significant changes, effect sizes varied across outcomes, and their clinical relevance remains uncertain and (vii) Several primary RCTs contributed to more than one pooled estimate for the same outcome (six trials for HbA1c, five for FBG, and one each for FPI, TG, TC, LDL‐C, and HDL‐C; Table S8). Consequently, pooled estimates that share primary RCTs are not independent, and agreement or disagreement among them should not be interpreted as independent replication of the evidence. For example, the HbA1c estimates reported by Cui et al. (2023), Jeong et al. (2022), and Chen et al. (2022) share two primary trials. Similarly, the lipid estimates reported by Chen et al. (2025) for comparisons with acarbose are based on a single trial that was also included in the pooled estimates of Chen et al. (2022). We also identified inconsistencies in how some shared trials were extracted or incorporated across meta‐analyses. For example, the HbA1c effect estimate from Asai et al. (2011) was reported as an MD of −1.10% in Chen et al. (2022), compared with 0.00% in Jeong et al. (2022) and Phimarn et al. (2017). In addition, Phimarn et al. (2017) appears to have counted participants from one trial Kim et al. (2012) twice within its FBG and HbA1c pooled estimates. These findings further limit the extent to which consistency across overlapping meta‐analyses can be interpreted as independent confirmation of an effect. Thus, the current evidence did not support definitive clinical recommendations.

### 4.2 Clinical Significance and Future Perspectives

The clinical relevance of the observed effects should be interpreted cautiously. The estimate in HbA1c (MD −0.51%, 95% CI −0.92 to −0.09) was derived from two RCTs comparing mulberry twig alkaloids to placebo in 181 adults with T2D with low‐certainty evidence. This finding should not be generalized to people without T2D. In contrast, the reductions in FBG (MD −34.23 mg/dL, 95% CI −55.13 to −13.33) and TG (MD −45.17 mg/dL, 95% CI −70.86 to −19.49) among patients with T2D may be clinically meaningful if confirmed in larger trials. However, both estimates were based on a single small randomized controlled trial (*n* = 30), and the wide CIs indicate considerable uncertainty. Across broader populations, the effects were smaller and less consistent. For example, TG decreased by 19.67 mg/dL and TC by 13.13 mg/dL, but these estimates were supported by very low‐ and low‐certainty evidence, respectively. Overall, these findings suggest that mulberry has the potential to improve glycemic control and TG levels, particularly in patients with T2D. However, small sample sizes and the very‐low‐to‐moderate certainty of the statistically significant findings prevent firm conclusions about the consistency and generalizability of these benefits.

According to this study, several important research gaps must be solved in order to promote translation of mulberry‐derived functional foods and supplements into clinical practice. First, further RCTs should not be limited to crude powders (leaf, fruit or twig) but use known standardized extracts with known levels of key bioactive compounds, particularly DNJ, oxyresveratrol, and anthocyanins. Such standardization is necessary for reproducibility, dose–response evaluation, and regulatory compliance, which may potentially translate into future clinical applications. Second, there is a need for diverse populations with more external validity of evidence. The majority of current trials have been conducted in Asian populations, and the findings that underlie such findings should not be generalizable. Large‐scale studies with non‐Asian populations and participants with a wider range of metabolic abnormalities ranging from early insulin resistance to established metabolic syndrome are required in order to better clarify population‐specific efficacy and safety. Third, formal subgroup analyses according to mulberry formulation, dose, treatment duration, and baseline metabolic status represent an important direction for future research. Because these analyses require trial‐level data, they are more appropriately conducted in original meta‐analyses of individual RCTs than in an umbrella review.

## 5 Conclusions

Our results indicated that consumption of different mulberry‐based preparations statistically modulated glycemic and lipid profiles. The observed declines in FBG, HbA1c, TG, TC, and LDL‐C suggested therapeutic potential for cardiometabolic management. However, the critically low methodological quality of the synthesized MAs and high statistical heterogeneity necessitate cautious interpretation. Moreover, because several of the statistically significant associations were supported only by low‐to‐very‐low certainty evidence under the GRADE framework, and all included meta‐analyses were rated as critically low in methodological quality by AMSTAR‐2, these findings should be regarded as preliminary and are insufficient to support definitive clinical recommendations. Standardized extracts and rigorous trial methodologies are required to set definitive clinical guidelines for future medical usage.

## Author Contributions

**Woorawee Inthachat:** conceptualization, methodology, investigation, validation, formal analysis, writing – original draft, writing – review and editing. **Chaowanee Chupeerach:** conceptualization, methodology, investigation, formal analysis, validation, writing – review and editing. **Teerapon Dhippayom:** methodology, resources, writing – review and editing, supervision, conceptualization, formal analysis, validation. **Piya Temviriyanukul:** conceptualization, methodology, validation, investigation, writing – original draft, writing – review and editing, formal analysis, supervision, data curation.

## Funding

No funding was received for this research. The article processing charge (APC) was paid by Mahidol University.

## Ethics Statement

This study was based solely on data extracted from previously published and publicly accessible meta‐analyses, thus no ethical approval or informed consent was required.

## Consent

This study was based solely on data extracted from previously published and publicly accessible meta‐analyses, thus no ethical approval or informed consent was required.

## Conflicts of Interest

The authors declare no conflicts of interest.

## Supporting information


**Table S1:** Comprehensive search strategy from CENTRAL database.
**Table S2:** Comprehensive search strategy from Embase database.
**Table S3:** Comprehensive search strategy from Epistemonikos database.
**Table S4:** Comprehensive search strategy from PubMed database.
**Table S5:** Comprehensive search strategy from Scopus database.
**Table S6:** Comprehensive search strategy from EBSCO Open Dissertations.
**Table S7:** Included and excluded meta‐analyses (MAs) of RCTs from article selection process.
**Table S8:** Cross‐tabulation of primary RCTs against the five included meta‐analyses (MAs), showing the outcomes to which each RCT contributed in the pooled estimates reported in Table 2.
**Table S9:** Quality assessment of included MAs using AMSTAR‐2.
**Table S10:** Certainty assessment of included MAs using GRADEpro GDT.

## Data Availability

Data were extracted from published meta‐analyses, all of which are accessible.

## References

[fsn372557-bib-0001] Abbas, M. A. , G. Y. Lee , S. A. J. Sayem , S. J. Lee , and S. C. Park . 2025. “Integrated Phytochemical Profiling, GC‐MS Characterization, and In Silico, In Vitro Evaluation of Synergistic Antimicrobial, Antioxidant, and Anti‐Inflammatory Activities of *Morus alba* Bark and *Pinus densiflora* Extracts With Methyl Gallate.” Antioxidants (Basel) 14, no. 9: 1114. 10.3390/antiox14091114. PMC12466517 41009018

[fsn372557-bib-0002] Ann, J. Y. , H. Eo , and Y. Lim . 2015. “Mulberry Leaves ( *Morus alba* L.) Ameliorate Obesity‐Induced Hepatic Lipogenesis, Fibrosis, and Oxidative Stress in High‐Fat Diet‐Fed Mice.” Genes & Nutrition 10, no. 6: 46. 10.1007/s12263-015-0495-x. PMC4604156 26463593

[fsn372557-bib-0003] Asai, A. , K. Nakagawa , O. Higuchi , et al. 2011. “Effect of Mulberry Leaf Extract With Enriched 1‐Deoxynojirimycin Content on Postprandial Glycemic Control in Subjects With Impaired Glucose Metabolism.” Journal of Diabetes Investigation 2, no. 4: 318–323. 10.1111/j.2040-1124.2011.00101.x. PMC4014974 24843505

[fsn372557-bib-0004] Bacha, F. , T. S. Hannon , M. Tosur , et al. 2024. “Pathophysiology and Treatment of Prediabetes and Type 2 Diabetes in Youth.” Diabetes Care 47, no. 12: 2038–2049. 10.2337/dci24-0029. PMC11655414 39250166

[fsn372557-bib-0005] Ballena‐Caicedo, J. , F. E. Zuzunaga‐Montoya , J. A. Loayza‐Castro , et al. 2025. “Global Prevalence of Dyslipidemias in the General Adult Population: A Systematic Review and Meta‐Analysis.” Journal of Health, Population, and Nutrition 44, no. 1: 308. 10.1186/s41043-025-01054-3. PMC12379389 40859400

[fsn372557-bib-0006] Caturano, A. , M. Rocco , G. Tagliaferri , et al. 2025. “Oxidative Stress and Cardiovascular Complications in Type 2 Diabetes: From Pathophysiology to Lifestyle Modifications.” Antioxidants 14, no. 1: 72. 10.3390/antiox14010072. PMC11759781 39857406

[fsn372557-bib-0007] Chawla, A. , R. Chawla , and S. Jaggi . 2016. “Microvasular and Macrovascular Complications in Diabetes Mellitus: Distinct or Continuum?” Indian Journal of Endocrinology and Metabolism 20, no. 4: 546–551. 10.4103/2230-8210.183480. PMC4911847 27366724

[fsn372557-bib-0008] Chen, G. , R. Yang , X. Yang , et al. 2025. “Mulberry Twig Alkaloids for Type 2 Diabetes Mellitus: A Systematic Review and Meta‐Analysis.” Frontiers in Pharmacology 16: 1475080. 10.3389/fphar.2025.1475080. PMC11903429 40083380

[fsn372557-bib-0009] Chen, X. , M. H. Sohouli , M. Nateghi , E. Melekoglu , and S. Fatahi . 2022. “Impact of Mulberry Consumption on Cardiometabolic Risk Factors: A Systematic Review and Meta‐Analysis of Randomized‐Controlled Trials.” Journal of Clinical Pharmacy and Therapeutics 47, no. 12: 1982–1993. 10.1111/jcpt.13822. 36509962

[fsn372557-bib-0010] Choi, G. J. , and H. Kang . 2023. “Introduction to Umbrella Reviews as a Useful Evidence‐Based Practice.” Journal of Lipid and Atherosclerosis 12, no. 1: 3–11. 10.12997/jla.2023.12.1.3. PMC9884555 36761061

[fsn372557-bib-0011] Cui, W. , K. Luo , Q. Xiao , et al. 2023. “Effect of Mulberry Leaf or Mulberry Leaf Extract on Glycemic Traits: A Systematic Review and Meta‐Analysis.” Food & Function 14, no. 3: 1277–1289. 10.1039/d2fo02645g. 36644880

[fsn372557-bib-0012] Duangjai, A. , K. Ingkaninan , and N. Limpeanchob . 2011. “Potential Mechanisms of Hypocholesterolaemic Effect of Thai Spices/Dietary Extracts.” Natural Product Research 25, no. 4: 341–352. 10.1080/14786411003754249. 20623425

[fsn372557-bib-0013] Gates, M. , A. Gates , D. Pieper , et al. 2022. “Reporting Guideline for Overviews of Reviews of Healthcare Interventions: Development of the PRIOR Statement.” BMJ 378: e070849. 10.1136/bmj-2022-070849. PMC9361065 35944924

[fsn372557-bib-0014] Guyatt, G. H. , A. D. Oxman , G. E. Vist , et al. 2008. “GRADE: An Emerging Consensus on Rating Quality of Evidence and Strength of Recommendations.” BMJ 336, no. 7650: 924–926. 10.1136/bmj.39489.470347.AD. PMC2335261 18436948

[fsn372557-bib-0015] Higgins, J. P. T. , J. Thomas , J. Chandler , M. Cumpston , T. Li , and M. J. Page . 2024. Cochrane Handbook for Systematic Reviews of Interventions Version 6.5 (Updated August 2024). Cochrane. https://www.cochrane.org/handbook. 10.1002/14651858.ED000142 PMC10284251 31643080

[fsn372557-bib-0016] Hirano, T. 2018. “Pathophysiology of Diabetic Dyslipidemia.” Journal of Atherosclerosis and Thrombosis 25, no. 9: 771–782. 10.5551/jat.RV17023. PMC6143775 29998913

[fsn372557-bib-0017] Huang, X. , Y. Wu , Y. Ni , H. Xu , and Y. He . 2025. “Global, Regional, and National Burden of Type 2 Diabetes Mellitus Caused by High BMI From 1990 to 2021, and Forecasts to 2045: Analysis From the Global Burden of Disease Study 2021.” Frontiers in Public Health 13: 1515797. 10.3389/fpubh.2025.1515797. PMC11798972 39916706

[fsn372557-bib-0018] Izzo, A. , R. Costanzo , L. De Martino , and S. D. Napoli . 2024. “Does EFSA Statement on Monacolin Content of Nutraceutical Combinations Impair Their Lipid Lowering Effect? The LopiGLIK Experience.” Clinical Diabetes and Endocrinology 10, no. 1: 38. 10.1186/s40842-024-00204-6. PMC11607800 39614370

[fsn372557-bib-0019] Jeong, H. I. , S. Jang , and K. H. Kim . 2022. “ *Morus alba* L. for Blood Sugar Management: A Systematic Review and Meta‐Analysis.” Evidence‐Based Complementary and Alternative Medicine 2022: 9282154. 10.1155/2022/9282154. PMC9152381 35656460

[fsn372557-bib-0020] Kim, H. J. , K. H. Yoon , M. J. Kang , et al. 2012. “A Six‐Month Supplementation of Mulberry, Korean Red Ginseng, and Banaba Decreases Biomarkers of Systemic Low‐Grade Inflammation in Subjects With Impaired Glucose Tolerance and Type 2 Diabetes.” Evidence‐Based Complementary and Alternative Medicine 2012: 735191. 10.1155/2012/735191. PMC3303547 22474520

[fsn372557-bib-0021] Kobayashi, Y. , M. Miyazawa , M. Araki , et al. 2015. “Effects of *Morus alba* L. (Mulberry) Leaf Extract in Hypercholesterolemic Mice on Suppression of Cholesterol Synthesis.” Journal of Pharmacognosy & Natural Products 1, no. 2: 113. 10.4172/jpnp.1000113.

[fsn372557-bib-0022] Liu, Q. , X. Li , C. Li , Y. Zheng , and G. Peng . 2015. “1‐Deoxynojirimycin Alleviates Insulin Resistance via Activation of Insulin Signaling PI3K/AKT Pathway in Skeletal Muscle of Db/Db Mice.” Molecules 20, no. 12: 21700–21714. 10.3390/molecules201219794. PMC6331926 26690098

[fsn372557-bib-0023] Morales Ramos, J. G. , A. T. Esteves Pairazamán , M. E. S. Mocarro Willis , S. Collantes Santisteban , and E. Caldas Herrera . 2021. “Medicinal Properties of *Morus alba* for the Control of Type 2 Diabetes Mellitus: A Systematic Review.” F1000Research 10: 1022. 10.12688/f1000research.55573.1. PMC8593624 34912543

[fsn372557-bib-0024] Nabrdalik, K. , K. Skonieczna‐Żydecka , K. Irlik , et al. 2022. “Gastrointestinal Adverse Events of Metformin Treatment in Patients With Type 2 Diabetes Mellitus: A Systematic Review, Meta‐Analysis and Meta‐Regression of Randomized Controlled Trials.” Frontiers in Endocrinology 13: 975912. 10.3389/fendo.2022.975912. PMC9524196 36187122

[fsn372557-bib-0025] Pacella, D. , M. Lembo , V. Trimarco , et al. 2025. “Dual Modulation of Lipid and Glucose Metabolism by a Nutraceutical Combination in Patients at Cardiometabolic Risk: Results From a Multicenter Randomized Controlled Trial.” Cardiovascular Diabetology 24, no. 1: 384. 10.1186/s12933-025-02920-4. PMC12495750 41044582

[fsn372557-bib-0026] Phimarn, W. , K. Wichaiyo , K. Silpsavikul , B. Sungthong , and K. Saramunee . 2017. “A Meta‐Analysis of Efficacy of *Morus alba* Linn. to Improve Blood Glucose and Lipid Profile.” European Journal of Nutrition 56, no. 4: 1509–1521. 10.1007/s00394-016-1197-x. 26972284

[fsn372557-bib-0027] Rao, H. , J. A. Jalali , T. P. Johnston , and P. Koulen . 2021. “Emerging Roles of Dyslipidemia and Hyperglycemia in Diabetic Retinopathy: Molecular Mechanisms and Clinical Perspectives.” Frontiers in Endocrinology 12: 620045. 10.3389/fendo.2021.620045. PMC8020813 33828528

[fsn372557-bib-0028] Shea, B. J. , B. C. Reeves , G. Wells , et al. 2017. “AMSTAR 2: A Critical Appraisal Tool for Systematic Reviews That Include Randomised or Non‐Randomised Studies of Healthcare Interventions, or Both.” BMJ 358: j4008. 10.1136/bmj.j4008. PMC5833365 28935701

[fsn372557-bib-0029] Srinivasa, U. M. , P. Kalaivani , and R. E. Anitha . 2026. “Chapter 14—Phytochemicals and Metabolic Disorders: Managing Diabetes, Obesity, and Metabolic Syndrome.” In Phytoceuticals in Food for Health and Wellness, edited by T. Sarkar , S. Smaoui , and W.‐F. Lai , 235–259. Academic Press. 10.1016/B978-0-443-26494-8.00004-5.

[fsn372557-bib-0030] Stancu, C. , and A. Sima . 2001. “Statins: Mechanism of Action and Effects.” Journal of Cellular and Molecular Medicine 5, no. 4: 378–387. 10.1111/j.1582-4934.2001.tb00172.x. PMC6740083 12067471

[fsn372557-bib-0031] Sun, H. , P. Saeedi , S. Karuranga , et al. 2022. “IDF Diabetes Atlas: Global, Regional and Country‐Level Diabetes Prevalence Estimates for 2021 and Projections for 2045.” Diabetes Research and Clinical Practice 183: 109119. 10.1016/j.diabres.2021.109119. PMC11057359 34879977

[fsn372557-bib-0032] Sun, Y. , X. Niu , Y. Wang , et al. 2025. “Effects of Mulberry Leaf and Corn Silk Extracts Against α‐Amylase and α‐Glucosidase In Vitro and on Postprandial Glucose in Prediabetic Individuals: A Randomized Crossover Trial.” Nutrients 17, no. 21: 3438. 10.3390/nu17213438. PMC12610157 41228506

[fsn372557-bib-0033] Suttisansanee, U. , S. Charoenkiatkul , B. Jongruaysup , S. Tabtimsri , D. Siriwan , and P. Temviriyanukul . 2020. “Mulberry Fruit Cultivar ‘Chiang Mai’ Prevents Beta‐Amyloid Toxicity in PC12 Neuronal Cells and in a Drosophila Model of Alzheimer's Disease.” Molecules 25, no. 8: 1837. 10.3390/molecules25081837. PMC7221829 32316271

[fsn372557-bib-0034] Temviriyanukul, P. , V. Sritalahareuthai , K. N. Jom , et al. 2020. “Comparison of Phytochemicals, Antioxidant, and In Vitro Anti‐Alzheimer Properties of Twenty‐Seven Morus spp. Cultivated in Thailand.” Molecules 25, no. 11: 2600. 10.3390/molecules25112600. PMC7321130 32503261

[fsn372557-bib-0035] Tsuduki, T. , Y. Nakamura , T. Honma , et al. 2009. “Intake of 1‐Deoxynojirimycin Suppresses Lipid Accumulation Through Activation of the β‐Oxidation System in Rat Liver.” Journal of Agricultural and Food Chemistry 57, no. 22: 11024–11029. 10.1021/jf903132r. 19863049

[fsn372557-bib-0036] Zhou, X. T. , A. Q. Zhu , X. M. Li , et al. 2024. “Mulberry and Hippophae‐Based Solid Beverage Promotes Weight Loss in Rats by Antagonizing White Adipose Tissue PPARγ and FGFR1 Signaling.” Frontiers in Endocrinology 15: 1344262. 10.3389/fendo.2024.1344262. PMC10978776 38559696

