# Supplementary material for: Impact of Mulberry Consumption on Glycemic Indices and Lipid Profiles: An Umbrella Review
Source: Food Sci Nutr. 2026 Nov 13;14(11):e72557. doi: 10.1002/fsn3.72557 (PMC13765480; doi:10.1002/fsn3.72557)
Supplement: Supplementary file 1 [file FSN3-14-e72557-s001.pdf]

Supporting Information

Impact of Mulberry Consumption on Glycemic Indices and Lipid Profiles: An Umbrella Review

Woorawee Inthachat [1], Chaowanee Chupeerach [1], Teerapon Dhippayom [2,3,4] and Piya Temviriyanukul [1, *]

1. Food and Nutrition Academic and Research Cluster, Institute of Nutrition, Mahidol University, Salaya, Phuttamonthon, Nakhon Pathom 73170, Thailand
2. The Research Unit of Evidence Synthesis, Faculty of Pharmaceutical Sciences, Naresuan University, Phitsanulok 65000, Thailand
3. Advancing Collaboration, Translation, and Innovation for Value in Evidence Synthesis (ACTIVES) Hub of Talent, Faculty of Pharmaceutical Sciences, Naresuan University, Phitsanulok 65000, Thailand
4. Department of Pharmacotherapy, College of Pharmacy, University of Utah, Salt Lake City, Utah 84112, USA

* Address correspondence to Institute of Nutrition, Mahidol University, Salaya, Phuttamonthon, Nakhon Pathom 73170, Thailand. Tel.: +662-800-2380 ext. 116; Fax: +662-441-9344. E-mail: piya.tem@mahidol.ac.th

Table S1: Comprehensive search strategy from CENTRAL database

Search number	Query	Results
#30	#27 AND #28 AND #29	0
#29	#24 OR #25 OR #26	23955
#28	#5 OR #6 OR #7 OR #8 OR #9 OR #10 OR #11 OR #12 OR #13 OR #14 OR #15 OR #16 OR #17 OR #18 OR #19 OR #20 OR #21 OR #22 OR #23	137947
#27	#1 OR #2 OR #3 OR #4	296
#26	MeSH descriptor: [Meta-Analysis as Topic] explode all trees	1494
#25	("meta analysis"):ti,ab,kw	23955
#24	(meta-analysis):ti,ab,kw	23955
#23	(lipid profile*):ti,ab,kw	16520
#22	MeSH descriptor: [Cholesterol] explode all trees	12818
#21	(cholesterol):ti,ab,kw	45136
#20	MeSH descriptor: [Triglycerides] explode all trees	7875
#19	(triglycerid*):ti,ab,kw	28841
#18	MeSH descriptor: [Lipoproteins, LDL] explode all trees	7414
#17	(LDL):ti,ab,kw	26766
#16	("low density lipoprotein"):ti,ab,kw	16459
#15	MeSH descriptor: [Cholesterol, HDL] explode all trees	4477
#14	("HDL cholesterol"):ti,ab,kw	7837
#13	("high density lipoprotein"):ti,ab,kw	12791
#12	MeSH descriptor: [Insulins] explode all trees	18482
#11	(insulin):ti,ab,kw	78740
#10	MeSH descriptor: [Glycated Hemoglobin] explode all trees	8489
#9	("HbA1c"):ti,ab,kw	26675
#8	("glycated haemoglobin"):ti,ab,kw	12453
#7	("postprandial glucose"):ti,ab,kw	3251
#6	MeSH descriptor: [Hypoglycemia] explode all trees	3165
#5	(fasting blood glucose):ti,ab,kw	24220
#4	MeSH descriptor: [Morus] explode all trees	58
#3	(Morus alba):ti,ab,kw	75
#2	(Morus):ti,ab,kw	135
#1	(mulberr*):ti,ab,kw	246

Table S2: Comprehensive search strategy from Embase database

Search number	Query	Results
#28	#25 AND #26 AND #27	11
#27	#23 OR #24	399,419
#26	#7 OR #8 OR #9 OR #10 OR #11 OR #12 OR #13 OR #14 OR #15 OR #16 OR #17 OR #18 OR #19 OR #20 OR #21 OR #22	1,196,341
#25	#1 OR #2 OR #3 OR #4 OR #5 OR #6	6,039
#24	'meta analysis'/exp AND topic	105,233
#23	'meta analysis':ti,ab,kw	381,446
#22	'lipid fingerprinting'/exp	28,345
#21	'lipid profile*':ti,ab,kw	73,325
#20	'triacylglycerol'/exp	299,594
#19	'triacylglycerol':ti,ab,kw	16,826
#18	'low density lipoprotein cholesterol'/exp	159,931
#17	'low density lipoprotein cholesterol':ti,ab,kw	44,352
#16	'high density lipoprotein cholesterol'/exp	158,425
#15	'high density lipoprotein':ti,ab,kw	81,959
#14	'insulin'/exp	442,065
#13	'insulin':ti,ab,kw	623,980
#12	'hemoglobin a1c'/exp	185,689
#11	'hemoglobin a1c':ti,ab,kw	22,790
#10	'glycated hemoglobin':ti,ab,kw	20,205
#9	'postpandial glucose':ti,ab,kw	5
#8	'fasting blood glucose level'/exp	14,906
#7	'fasting blood glucose level':ti,ab,kw	1,988
#6	'mulberry extract'/exp	1,131
#5	'morus alba'/exp	1,260
#4	'morus'/exp	2,504
#3	'morus':ti,ab,kw	2,639
#2	'morus alba':ti,ab,kw	1,609
#1	mulberr*:ti,ab,kw	4,012

Table S3: Comprehensive search strategy from Epistemonikos database

Search number	Query	Results
#5	(title:(mulberr*) OR abstract:(mulberr*)) OR (title:(morus) OR abstract:(morus)) OR (title:(morus alba) OR abstract:(morus alba)) AND (title:(meta-analysis) OR abstract:(meta-analysis))	3
#4	(title:(meta-analysis) OR abstract:(meta-analysis))	306,971
#3	(title:(morus alba) OR abstract:(morus alba))	126
#2	(title:(morus) OR abstract:(morus))	180
#1	(title:(mulberr*) OR abstract:(mulberr*))	376

Table S4: Comprehensive search strategy from PubMed database

Search number	Query	Results
29	(((((mulberr*[Title/Abstract]) OR ("morus"[Title/Abstract])) OR ("Morus alba"[Title/Abstract])) OR ("Morus alba" [Supplementary Concept] OR "Morus"[Mesh])) AND ((((((((((((((((((((fasting blood glucose[Title/Abstract]) OR ("Hypoglycemia"[Mesh])) OR (postprandial glucose[Title/Abstract])) OR (glycated haemoglobin[Title/Abstract])) OR (HbA1c[Title/Abstract])) OR ("Glycated Hemoglobin"[Mesh])) OR ("insulin"[Title/Abstract])) OR ("Insulin"[Mesh])) OR ("high density lipoprotein"[Title/Abstract])) OR ("HDL"[Title/Abstract])) OR ("Lipoproteins, HDL"[Mesh])) OR ("low density lipoprotein"[Title/Abstract])) OR ("LDL"[Title/Abstract])) OR ("Lipoproteins, LDL"[Mesh])) OR (triglycerid*[Title/Abstract])) OR ("Triglycerides"[Mesh])) OR ("cholesterol"[Title/Abstract])) OR ("Cholesterol"[Mesh])) OR (lipid profile*[Title/Abstract]))))) AND (("meta-analysis"[Title/Abstract]) OR ("Meta-Analysis" [Publication Type]))	6
28	("meta-analysis"[Title/Abstract]) OR ("Meta-Analysis" [Publication Type])	328,668
27	((((((((((((((((((fasting blood glucose[Title/Abstract]) OR ("Hypoglycemia"[Mesh])) OR (postprandial glucose[Title/Abstract])) OR (glycated haemoglobin[Title/Abstract])) OR (HbA1c[Title/Abstract])) OR ("Glycated Hemoglobin"[Mesh])) OR ("insulin"[Title/Abstract])) OR ("Insulin"[Mesh])) OR ("high density lipoprotein"[Title/Abstract])) OR ("HDL"[Title/Abstract])) OR ("Lipoproteins, HDL"[Mesh])) OR ("low density lipoprotein"[Title/Abstract])) OR ("LDL"[Title/Abstract])) OR ("Lipoproteins, LDL"[Mesh])) OR (triglycerid*[Title/Abstract])) OR ("Triglycerides"[Mesh])) OR ("cholesterol"[Title/Abstract])) OR ("Cholesterol"[Mesh])) OR (lipid profile*[Title/Abstract])	961,407
26	(((mulberr*[Title/Abstract]) OR ("morus"[Title/Abstract])) OR ("Morus alba"[Title/Abstract])) OR ("Morus alba" [Supplementary Concept] OR "Morus"[Mesh])	4,608
25	"Meta-Analysis" [Publication Type]	218,620
24	"meta-analysis"[Title/Abstract]	301,332
23	lipid profile*[Title/Abstract]	44,830
22	"Cholesterol"[Mesh]	180,522
21	"cholesterol"[Title/Abstract]	303,474
20	"Triglycerides"[Mesh]	88,940
19	triglycerid*[Title/Abstract]	147,945
18	"Lipoproteins, LDL"[Mesh]	62,738
17	"LDL"[Title/Abstract]	98,155
16	"low density lipoprotein"[Title/Abstract]	87,791
15	"Lipoproteins, HDL"[Mesh]	49,778
14	"HDL"[Title/Abstract]	83,769
13	"high density lipoprotein"[Title/Abstract]	65,328
12	"Insulin"[Mesh]	205,430
11	"insulin"[Title/Abstract]	437,660
10	"Glycated Hemoglobin"[Mesh]	47,315

9	HbA1c[Title/Abstract]	59,086
8	glycated haemoglobin[Title/Abstract]	5,551
7	postprandial glucose[Title/Abstract]	4,654
6	"Hypoglycemia"[Mesh]	33,084
5	fasting blood glucose[Title/Abstract]	19,227
4	"Morus alba" [Supplementary Concept] OR "Morus"[Mesh]	2,097
3	"Morus alba"[Title/Abstract]	1,290
2	"morus"[Title/Abstract]	2,158
1	mulberr*[Title/Abstract]	3,521

Table S5: Comprehensive search strategy from Scopus database

Search number	Query	Results
#18	((TITLE-ABS-KEY (morus AND alba)) OR (TITLE-ABS-KEY (morus)) OR (TITLE-ABS-KEY (mulberr*))) AND ((TITLE-ABS-KEY (meta AND analysis)) OR (TITLE-ABS-KEY (meta-analysis))) AND ((TITLE-ABS-KEY (fasting AND blood AND glucose)) OR (TITLE-ABS-KEY (postprandial AND glucose)) OR (TITLE-ABS-KEY (glycated AND haemoglobin)) OR (TITLE-ABS-KEY (hba1c)) OR (TITLE-ABS-KEY (insulin)) OR (TITLE-ABS-KEY (high AND density AND lipoprotein)) OR (TITLE-ABS-KEY (low AND density AND lipoprotein)) OR (TITLE-ABS-KEY (triglyceride*)) OR (TITLE-ABS-KEY (lipid AND profile*)))	22
#17	(TITLE-ABS-KEY (meta AND analysis)) OR (TITLE-ABS-KEY (meta-analysis))	569,986
#16	(TITLE-ABS-KEY fasting AND blood AND glucose)) OR TITLE-ABS-KEY (postprandial AND glucose)) OR (TITLE-ABS-KEY (glycated AND haemoglobin)) OR (TITLE-ABS-KEY (hba1c)) OR (TITLE-ABS-KEY (insulin)) OR (TITLE-ABS-KEY (high AND density AND lipoprotein)) OR (TITLE-ABS-KEY (low AND density AND lipoprotein)) OR (TITLE-ABS-KEY (triglyceride*)) OR (TITLE-ABS-KEY (lipid AND profile*))	1,382,591
#15	(TITLE-ABS-KEY (morus AND alba)) OR (TITLE-ABS-KEY (morus)) OR (TITLE-ABS-KEY (mulberr*))	15,003
#14	TITLE-ABS-KEY (meta-analysis)	513,178
#13	TITLE-ABS-KEY (meta AND analysis)	569,986
#12	TITLE-ABS-KEY (lipid AND profile*)	124,840
#11	TITLE-ABS-KEY (triglyceride*)	223,390
#10	TITLE-ABS-KEY (low AND density AND lipoprotein)	275,079
#9	TITLE-ABS-KEY (high AND density AND lipoprotein)	241,671
#8	TITLE-ABS-KEY (insulin)	929,447
#7	TITLE-ABS-KEY (hba1c)	65,404
#6	TITLE-ABS-KEY (glycated AND haemoglobin)	38,539
#5	TITLE-ABS-KEY (postprandial AND glucose)	24,291
#4	TITLE-ABS-KEY (fasting AND blood AND glucose)	108,573
#3	TITLE-ABS-KEY (mulberr*)	12,195
#2	TITLE-ABS-KEY (morus)	8,243
#1	TITLE-ABS-KEY (morus AND alba)	3,680

Table S6: Comprehensive search strategy from EBSCO Open Dissertations

Search number	Query	Results
#16	S13 AND S14 AND S15	0
#15	S4 OR S5 OR S6 OR S7 OR S8 OR S9 OR S10 OR S11 OR S12	14,471
#14	S1 OR S2 OR S3	51
#13	TI meta-analysis OR AB meta-analysis	4,595
#12	TI triglycerides OR AB triglycerides	1,451
#11	TI lipid profile OR AB lipid profile	1,485
#10	TI cholesterol OR AB cholesterol	3,484
#9	TI (ldl or low density lipoprotein or cholesterol) OR AB (ldl or low density lipoprotein or cholesterol)	4,092
#8	TI (hdl cholesterol levels or hdl cholesterol or high density lipoprotein) OR AB (hdl cholesterol levels or hdl cholesterol or high density lipoprotein)	935
#7	TI high density lipoprotein OR AB high density lipoprotein	629
#6	TI insulin OR AB insulin	6,338
#5	TI (hba1c or glycaemic control or glycemic control or blood sugar or glucose level) OR AB (hba1c or glycaemic control or glycemic control or blood sugar or glucose level)	4,814
#4	TI fasting blood glucose OR AB fasting blood glucose	421
#3	TI mulberry OR AB mulberry	47
#2	TI morus alba OR AB morus alba	3
#1	TI morus AND AB morus	5

Table S7: Included and excluded meta-analyses (MAs) of RCTs from article selection process.

**MAs identified from Database search**				
No.	Title	Author and year	DOI	Included or Excluded
1	Mulberry twig alkaloids for type 2 diabetes mellitus: a systematic review and meta-analysis	Chen 2025	https://doi.org/10.3389/fphar.2025.1475080	Included
2	Effect of mulberry leaf or mulberry leaf extract on glycemic traits: a systematic review and meta-analysis	Cui 2023	https://doi.org/10.1039/D2FO02645G	Included
3	Impact of mulberry consumption on cardiometabolic risk factors: A systematic review and meta-analysis of randomized-controlled trials	Chen 2022	https://doi.org/10.1111/jcpt.13822	Included
4	*Morus alba* L. for Blood Sugar Management: A Systematic Review and Meta-Analysis	Jeong 2022	https://doi.org/10.1155/2022/9282154	Included
5	A meta-analysis of efficacy of *Morus alba* Linn. to improve blood glucose and lipid profile	Phimarn 2017	https://doi.org/10.1007/s00394-016-1197-x	Included
6	Effects of 1-Deoxynojirimycin on glycemic control: A systematic review and meta-analysis	Cho 2025	https://doi.org/10.1016/j.nfs.2024.100210	Exclude (no intervention or outcomes of interest)
7	Effect of Berry Polyphenols on Glucose Metabolism: A Systematic Review and Meta-Analysis of Randomized Controlled Trials	Rambaran 2020	https://doi.org/10.1093/cdn/nzaa100	Exclude (no intervention or outcomes of interest)
**MAs identified from other search methods**				
1	Effect of anthocyanin supplementation on cardio-metabolic biomarkers: A systematic review and meta-analysis of randomized controlled trials	Daneshzad 2019	https://doi.org/10.1016/j.clnu.2018.06.979	Exclude (no intervention or outcomes of interest)

Table S8. Cross-tabulation of primary RCTs against the five included meta-analyses (MAs), showing the outcomes to which each RCT contributed in the pooled estimates reported in Table 2.

Primary RCT (first author, year)	Chen 2025	Chen 2022	Cui 2023	Jeong 2022	Phimarn 2017	No. of MAs including the RCT	Outcomes shared by ≥2 MAs
Asai 2011		1, 2, 3		1, 2	1, 2	3	**1, 2**
Riche 2017		1	1, 2	1		3	**1**
Thaipitakwong 2020[e]		1, 2, 3	1, 2, 3	1, 2		3	**1, 2, 3**
Kim JY 2015[d]			1, 2, 3	R	R	3	–
Kimura 2007			2	R	R	3	–
Li 2016	1, 2, 4, 5, 6, 7	1, 2, 4, 5, 6, 7				2	**1, 2, 4, 5, 6, 7**
Andallu 2001		1, 2, 4, 5, 6, 7	1, 2			2	**1, 2**
Aramwit 2011[b]		4, 5, 6, 7	1, 2			2	–
Trimarco 2015			1, 2, 3		1, 2, 4, 5, 6, 7	2	**1, 2**
Chen 2016	1, 2, 4, 5					1	–
Qu 2021	1, 2					1	–
Qu 2022	1					1	–
Yang & Ning 2023 (A)[f]	2					1	–
Aramwit 2013		4, 5, 6, 7				1	–
Sirikanchanarod 2016		4, 5, 6, 7				1	–
Taghizadeh 2017[c]		2, 3, 4, 5, 6, 7				1	–
Wang 2020		4, 5, 6, 7				1	–
Kalman 2017		R				1	–
Ma 2019		R				1	–
Kojima 2010			1, 2, 3			1	–
Qi 2018			1, 2			1	–
Taghizadeh 2022			1, 2, 3			1	–
Yang 2019			1, 2			1	–
Yasumoto 2022			1, 2			1	–
Yang & Han 2006				1, 2		1	–
Banchobphutsa 2014					2, 4, 5, 6, 7	1	–
Banu 2015					R	1	–
Choonwatchana 2015					2, 4, 5, 6, 7	1	–
Chung 2013					R	1	–
Hu 2014					1, 2, 4, 5, 6, 7	1	–
Kim 2012					1, 2[a]	1	–
Mudra 2007					R	1	–
Nakamura 2011					R	1	–
Sukriket 2014					R	1	–
**RCTs included in each MA (n)**	**5**	**12**	**12**	**6**	**13**	**48**	
**RCTs contributing to ≥1 estimate in Table 2 (n)**	**5**	**10**	**12**	**4**	**6**	**37**	

Numbers indicate the outcomes for which the RCT contributed data to that MA's pooled estimate reported in Table 2: 1, HbA1c; 2, FBG; 3, FPI; 4, TG; 5, TC; 6, LDL-C; 7, HDL-C. R, included in the MA but did not contribute to any pooled estimate reported in Table 2 (e.g., contributed only to iAUC, blood pressure or CRP analyses). A blank cell indicates that the RCT was not included in that MA. "Outcomes shared by ≥2 MAs" lists the outcomes for which the RCT contributed to the pooled estimates of two or more MAs; –, none.

Overall, the five MAs comprised 48 RCT inclusions corresponding to 34 unique RCTs; 9 RCTs were included in more than one MA, of which 6 contributed to the pooled estimates of the same outcome in two or more MAs.

For Chen 2025, Chen 2016 and Qu 2022 contribute to study A (mulberry twig alkaloids vs placebo), and Li 2016, Qu 2021 and Yang & Ning 2023 (A) contribute to study B (mulberry twig alkaloids vs acarbose or metformin) in Table 2.

[a] Phimarn (2017) entered the 12- and 24-week results of Kim (2012) as separate comparisons in its FBG and HbA1c analyses. [b] Labeled "Aramwit (2010)" in Chen 2022. [c] Labeled "Bahmani (2017)" in the forest plots of Chen 2022. [d] Labeled "Kim (2014)" in Jeong 2022. [e] Dated 2019 in Chen 2022 and Jeong 2022. [f] Arm A only (mulberry twig alkaloids vs metformin); arm B (mulberry twig alkaloids plus metformin vs metformin) was not included in this umbrella review.

Abbreviations: CRP, C-reactive protein; FBG, fasting blood glucose; FPI, fasting plasma insulin; HbA1c, glycated hemoglobin; HDL-C, high-density lipoprotein cholesterol; iAUC, incremental area under the curve; LDL-C, low-density lipoprotein cholesterol; MA, meta-analysis; RCT, randomized controlled trial; TC, total cholesterol; TG, triglycerides.

Table S9. Quality assessment of included MAs using AMSTAR-2.

Study	Q1	Q2	Q3	Q4	Q5	Q6	Q7	Q8	Q9	Q10	Q11	Q12	Q13	Q14	Q15	Q16	Quality assessment
Chen 2025	Yes	Yes	Yes	PY	Yes	Yes	No	Yes	Yes	Yes	Yes	No	No	Yes	Yes	Yes	Critically Low
Cui 2023	Yes	PY	Yes	No	Yes	Yes	No	Yes	Yes	No	Yes	No	No	Yes	Yes	Yes	Critically Low
Chen 2022	Yes	PY	Yes	PY	No	Yes	No	Yes	Yes	No	Yes	No	No	Yes	Yes	Yes	Critically Low
Jeong 2022	Yes	PY	Yes	PY	Yes	Yes	No	Yes	Yes	Yes	Yes	No	No	Yes	Yes	Yes	Critically Low
Phimarn 2017	Yes	No	Yes	PY	Yes	Yes	No	PY	Yes	No	Yes	No	No	Yes	Yes	Yes	Critically Low

Abbreviation: PY: partial yes; **Remarks**: Grey-shaded columns indicate the critical questions.

Table S10. Certainty assessment of included MAs using GRADEpro GDT.

Certainty assessment								95% CI	Certainty
Author	No. of studies	Study design	Risk of bias	Inconsistency	Indirectness	Imprecision	Other considerations		
**HbA1c**									
Chen 2025 (study A)	2	randomised trials	serious	serious	not serious	not serious	none	MD 0.51 % lower (0.92 lower to 0.09 lower)	⨁⨁◯◯ Low
Chen 2025 (study B)	2	randomised trials	not serious	not serious	not serious	very serious	none	MD 0.06 % lower (0.21 lower to 0.1 higher)	⨁⨁◯◯ Low
Cui 2023	11	randomised trials	serious	very serious	not serious	not serious	none	MD 0.27 % lower (0.52 lower to 0.02 lower)	⨁◯◯◯ Very low
Chen 2022	5	randomised trials	serious	very serious	serious	not serious	none	MD 0.55 % lower (1.08 lower to 0.02 lower)	⨁◯◯◯ Very low
Jeong 2022	4	randomised trials	serious	not serious	not serious	not serious	none	MD 0.05 % lower (0.18 lower to 0.08 higher)	⨁⨁⨁◯ Moderate
Phimarn 2017	5	randomised trials	not serious	not serious	not serious	very serious	none	MD 0.05 % lower (0.19 lower to 0.09 higher)	⨁⨁◯◯ Low
**FBG**									
Chen 2025 (study A)	1	randomised trials	serious	not serious	not serious	not serious	none	MD 34.23 mg/dL lower (55.13 lower to 13.33 lower)	⨁⨁⨁◯ Moderate
Chen 2025 (study B)	3	randomised trials	serious	not serious	not serious	very serious	none	MD 1.26 mg/dL lower (7.21 lower to 4.68 higher)	⨁◯◯◯ Very low
Cui 2023	12	randomised trials	serious	very serious	not serious	not serious	none	MD 8.47 mg/dL lower (15.13 lower to 1.80 lower)	⨁◯◯◯ Very low
Chen 2022	5	randomised trials	serious	serious	serious	serious	none	MD 7.5 mg/dL lower (19.58 lower to 4.59 higher)	⨁◯◯◯ Very low
Jeong 2022	3	randomised trials	serious	not serious	not serious	very serious	none	MD 0.79 mg/dL lower (4.56 lower to 2.98 higher)	⨁◯◯◯ Very low
Phimarn 2017	7	randomised trials	serious	serious	not serious	serious	none	MD 4.50 mg/dL lower (9.91 lower to 0.90 higher)	⨁◯◯◯ Very low
**FPI**									
Chen 2022	3	randomised trials	serious	not serious	serious	very serious	none	MD 0.13 higher (1.29 lower to 1.55 higher)	⨁◯◯◯ Very low
Cui 2023	5	randomised trials	serious	serious	not serious	very serious	none	MD 0.58 µIU/mL lower (2.78 lower to 1.62 higher)	⨁◯◯◯ Very low
**Triglyceride**									
Chen 2025 (study A)	1	randomised trials	serious	not serious	not serious	not serious	none	MD 45.17 mg/dL lower (70.86 lower to 19.49 lower)	⨁⨁⨁◯ Moderate
Chen 2025 (study B)	1	randomised trials	not serious	not serious	not serious	very serious	none	MD 18.60 mg/dL lower (81.49 lower to 44.29 higher)	⨁⨁◯◯ Low
Chen 2022	7	randomised trials	serious	seriousb	seriousd	not serious	none	MD 19.67 mg/dL lower (30.13 lower to 9.22 lower)	⨁◯◯◯ Very low

Certainty assessment								95% CI	Certainty
Author	No. of studies	Study design	Risk of bias	Inconsistency	Indirectness	Imprecision	Other considerations		
Phimarn 2017	4	randomised trials	not serious	not serious	not serious	not serious	none	MD 17.71 mg/dL lower (37.20 lower to 1.77 higher)	⊕⊕⊕⊕ High
**Cholesterol**									
Chen 2025 (study A)	1	randomised trials	serious	not serious	not serious	serious	none	MD 10.83 mg/dL lower (30.16 lower to 8.51 higher)	⊕⊕◯◯ Low
Chen 2025 (study B)	1	randomised trials	not serious	not serious	not serious	very serious	none	MD 2.71 mg/dL higher (23.20 lower to 28.62 higher)	⊕⊕◯◯ Low
Chen 2022	7	randomised trials	serious	not serious	serious	not serious	none	MD 13.13 mg/dL lower (19.06 lower to 7.20 lower)	⊕⊕◯◯ Low
Phimarn 2017	4	randomised trials	not serious	serious	not serious	not serious	none	MD 6.57 mg/dL lower (23.98 lower to 10.44 higher)	⊕⊕⊕◯ Moderate
**LDL-C**									
Chen 20205	1	randomised trials	not serious	not serious	not serious	serious	none	MD 5.80 mg/dL lower (22.04 lower to 10.44 higher)	⊕⊕⊕◯ Moderate
Chen 2022	7	randomised trials	serious	not serious	serious	not serious	none	MD 8.84 mg/dL lower (13.26 lower to 4.42 lower)	⊕⊕◯◯ Low
Phimarn 2017	4	randomised trials	not serious	serious	not serious	very serious[f]	none	MD 4.25 mg/dL lower (20.11 lower to 11.60 higher)	⊕◯◯◯ Very low
**HDL-C**									
Chen 2025	1	randomised trials	not serious	not serious	not serious	not serious	none	MD 2.32 mg/dL lower (6.96 lower to 2.32 higher)	⊕⊕⊕⊕ High
Chen 2022	7	randomised trials	serious	not serious	serious	not serious	none	MD 1.51 mg/dL higher (0.40 lower to 3.42 higher)	⊕⊕◯◯ Low
Phimarn 2017	4	randomised trials	not serious	serious	not serious	very serious	none	MD 0.00 mg/dL (3.87 lower to 4.25 higher)	⊕◯◯◯ Very low
